# Factors related to parental pre-treatment motivation in outpatient child and adolescent mental health care

**DOI:** 10.1007/s00787-019-01391-9

**Published:** 2019-09-24

**Authors:** Halewijn M. Drent, Barbara van den Hoofdakker, Annelies de Bildt, Jan K. Buitelaar, Pieter J. Hoekstra, Andrea Dietrich

**Affiliations:** 1 Department of Child and Adolescent Psychiatry, University Medical Center Groningen, University of Groningen, Hanzeplein 1 A10, 9700 GZ Groningen, The Netherlands; 2 Department of Cognitive Neuroscience, Donders Institute for Brain Cognition and Behaviour, Radboud University Medical Center, Nijmegen, The Netherlands

**Keywords:** Parental pre-treatment motivation, Child and adolescent psychiatry, LASSO regression, Behavioural problems, Emotional problems

## Abstract

The aim of this study was to investigate the relation between a variety of child, parent, family and environmental factors and pre-treatment motivation of parents of children and adolescents newly referred to a mental health care clinic in The Netherlands. Data were collected of 521 parents most involved in the upbringing of the child (443 mothers and 78 fathers; Dutch origin 97.1%) of 207 girls and 314 boys (age *M* = 10.2, range 1–18 years). Treatment motivation was measured by the Parent Motivation Inventory. Least absolute shrinkage and selection operator (LASSO) regression was used to investigate the prediction strength of 33 factors in 5 domains: (1) source of referral and prior use of healthcare services, (2) child characteristics, (3) characteristics of the primary parent, (4) parenting characteristics of the primary parent, and (5) family characteristics. Twenty-one factors were statistically relevant, explaining 21.3% of the deviance in pre-treatment motivation. Child characteristics, mainly type and severity of problems, contributed most to the model. Notably, internalising problems contributed more than externalising problems. Furthermore, we found relations between parental pre-treatment motivation and parents' perceived self-efficacy, parents' perceived parenting competence, financial problems and source of referral. Our findings provide insight into the multifacetedness of parental motivation prior to starting treatment and inform health professionals of specific contextual factors of interest in parents' readiness to change their behaviour and participate in treatment.

**Electronic supplementary material:**

The online version of this article (10.1007/s00787-019-01391-9) contains supplementary material, which is available to authorized users.

## Introduction

Parental motivation for treatment in child and adolescent mental health outpatient clinics is increasingly recognised as an important prerequisite for treatment success and for preventing treatment dropout [1–7]. Treatment motivation may be conceptualized as readiness to change one’s behaviour and willingness to participate in treatment [1, 6, 8, 9], but also facilitating the child in getting to appointments or using prescriptions [10]. A number of studies have focused on engagement of parents during treatment (e.g., barriers to treatment, attendance rates, or dropout) [11–13]. Especially the transtheoretical model of behavior change [14, 15] shows the different ways in which treatment engagement may change throughout the help-seeking and help-receiving process and, in addition, the importance of treatment engagement in child and adolescent mental healthcare [16]. However, little attention has been given to pre-treatment motivation, defined as parental readiness to start treatment (e.g., readiness to change one’s behaviour, desire for change, perceived ability to change), and to the individual (e.g., patient’s gender, age or problem severity) and contextual factors (e.g., source of referral, parental characteristics, social network strength) that are related to it. Addressing these individual and contextual factors may foster the identification of particularly vulnerable families characterised by lower motivation for treatment in child and adolescent mental health care and facilitate tailored interventions to increase treatment motivation in those families who are newly referred to a child and adolescent outpatient mental health clinic for emotional and/or behavioural problems of their child.

Previous studies identified a number of parent characteristics associated with parents’ pre-treatment motivation. In a study among 386 families who took part in an intervention for children at risk for behavioural problems, a higher level of parental depression was related to higher levels of motivation (i.e., desire to change) of these parents to change their parenting techniques [17]. Also, higher parental stress was related to a higher level of parental pre-treatment motivation (i.e., treatment readiness, readiness to change) [18, 19]. Furthermore, poorer parenting skills (i.e., higher levels of inconsistent disciplining and poor supervision) were associated with higher parental motivation (i.e., treatment readiness) for receiving parenting treatment for parents of children with behavioural problems [20].

These studies also investigated child factors in relation to parents’ pre-treatment motivation. Higher symptom severity was found to be related to higher parental pre-treatment motivation, as reported in a study among 197 adolescents when entering home-based treatment services for a variety of mental health problems [18] and in two studies of children who were referred to a mental health clinic for behavioural problems [19, 20].

The sparse literature about factors related to parents’ treatment motivation prior to starting treatment in child and adolescent mental health care has important shortcomings: studies often consisted of small sample sizes, considered only a limited number of factors and focused predominantly on children with externalising problem behaviour rather than on the broad scope of problems of children and adolescents referred to mental health services [3, 17, 19–21]. Also, weaknesses in the various measures of motivation for treatment may be noted, e.g., rating scales with low internal consistency, a low number of items or open-ended questions [3, 17, 21].

In the current study, we addressed the relation of a wide range of individual (i.e., child and parent) and contextual factors (i.e., family and environmental factors) with parents’ pre-treatment motivation in a large sample of children and adolescents newly referred to one of the participating child and adolescent outpatient mental health clinics in this study. To assess parental pre-treatment motivation, we used the composite score of a validated parent rating scale that conceptualizes three aspects of motivation: desire for change, readiness to change and perceived ability to change [5]. Potential predictors were divided into five domains: (1) source of referral and prior use of health services, (2) child characteristics, (3) characteristics of the primary parent, (4) parenting characteristics of the primary parent and (5) family characteristics. In line with abovementioned studies, we expected higher parental stress and worse parental mental health (i.e., depression and anxiety), poorer parenting techniques and more severe child problems to be related to higher parental pre-treatment motivation. Furthermore, we investigated whether other individual and contextual factors were related to parental pre-treatment motivation.

## Method

### Participants

Our study included 521 families who participated in the baseline assessment of a three-wave survey study on the influence of child, family and environmental factors on response to treatment in outpatient child and adolescent mental health services. Families of children up to age 18 years (97.1% with the primary parent of Dutch origin) were recruited from two large child and adolescent psychiatry centres with several locations in the Northern and Eastern part of the Netherlands, including both rural and urban areas. Children had to be newly referred (i.e., first referral to the respective mental healthcare clinic) to be eligible to participate in the study. Children referred for eating disorders or forensic problems were not invited to the study, because treatment trajectories in these settings are not always entirely voluntary, making pre-treatment motivation different from voluntary participants. The primary parent (i.e., the parent most involved in raising the child) and the child (≥ 8 years old) were asked to fill out a set of questionnaires at each of the three waves related to a variety of contextual factors. Completion of questionnaires by the secondary parent (i.e., the partner of the primary parent living in the same household; in 82.9% of the families this was a biological parent of the child) was optional and only at baseline. In the present study, we used the baseline data of the primary parent, except for socio-economic status which also included data provided by the secondary parent. Participation was voluntary and families were rewarded with a voucher of 20 euros for participation in each study wave. It should be noted that mental healthcare in the Netherlands is accessible to all families. There were no incentives for a family to follow treatments, which were all voluntary.

### Procedures

**Fig. 1:**
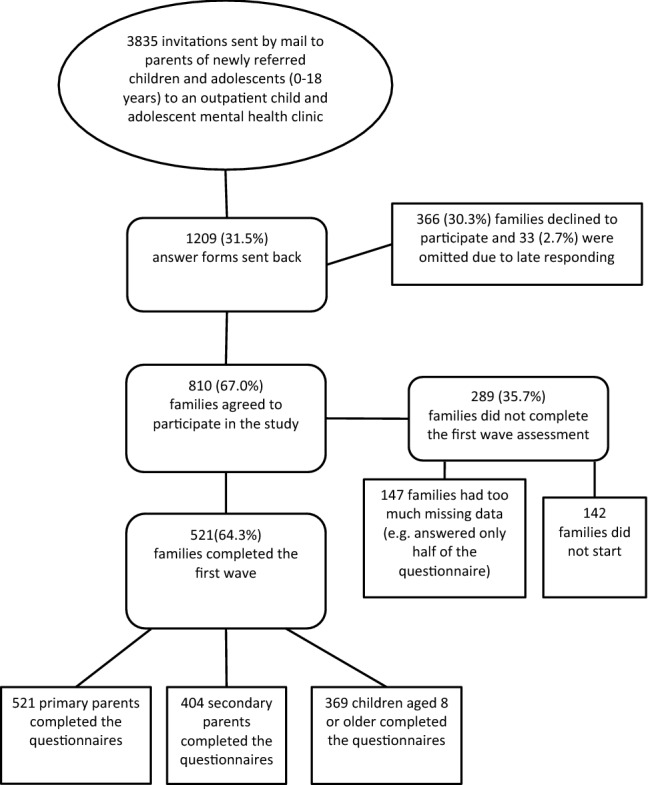
Participation flow of the baseline assessment of a three-wave study on the influence of child, family and environmental factors on response to treatment in outpatient child and adolescent mental healthcare

Figure 1 describes the sampling procedure of the baseline assessment. An invitation to participate in the study was sent along with the invitation for the first clinic visit by mail to all newly referred families between 1st May 2015 and 30th June 2016. Families who agreed to participate received personal login codes for the primary parent, secondary parent and child (≥ 8 years old) to a set of online questionnaires via mail before a possible start of treatment. Twenty-one families received a paper version of the questionnaires. On average, families completed the questionnaire within 18 days after the personal login codes were sent (with a range of 1 day–3 months). E-mail reminders to respond to our invitation were sent 2 and 3 weeks after the personal login codes were sent. Families who still did not respond after the second e-mail reminder were approached by telephone (at least three calls made on different days and times). Families who agreed to participate or started the questionnaire after the 30th of June 2016 were omitted from the sample. Based on information from the electronic data entry system, completion of the baseline questionnaires took the primary parents about 75 min (*n* = 512), the secondary parent about 30 min (*n* = 404) and the child about 20 min (*n* = 369). Participants were able to pause answering the online questionnaire, but they were asked to complete the full set of questionnaires within 1 week after they started. Lastly, families were asked to complete the questionnaires before a possible start of treatment at the outpatient centres.

### Measures

#### Outcome measure

Parental pre-treatment motivation was measured by the Parent Motivation Inventory (PMI), showing good validity and reliability [5, 22]. Parents rated 25 items on a five-point scale (1 = strongly disagree, 5 = strongly agree; observed Cronbach’s *α* = 0.93) addressing the need to change (e.g., ‘My child’s behaviour has to improve soon’), readiness to change (‘I am willing to work on changing my own behaviour as it relates to managing my child’) and perceived ability to change (‘I believe that my child’s behaviour cannot change without my involvement in treatment’); note that one item was removed from the scale (i.e., ‘I am motivated to change the way I reward and punish my child if it will lead to improvement’), because there were too many missing responses on this item due to a technical difficulty in the online questionnaire. The PMI is a valid measure tested in a clinical sample of parents of American children with externalising problem behaviour (*α* = 0.86) [5]. Furthermore, construct validity of the PMI is tested by the correlation with the credibility and expectancy questionnaire [22]. A higher score on the PMI indicates a higher level of parental pre-treatment motivation.

#### Predictors

Predictors of parental pre-treatment motivation were distinguished in five domains as described below. We used the total scale scores of questionnaires where appropriate. A detailed description of the scales can be found in Online Resource 1. Only data of the primary parents were used, unless otherwise noted.

Source of referral and prior use of healthcare services was measured by asking the primary parent who had initiated the help-seeking process: (1) parent(s) or child, (2) school, or (3) a health professional (e.g., general practitioner or hospital consultant). To assess the child’s prior use of mental health services, the primary parent was asked whether the child had received some form of prior help for emotional and/or behavioural problems. Also, we assessed current medication use of the child via an open-ended question. Answers were dichotomised into ‘No medication use’ and ‘Medication use’; both non-psychotropic as well as psychotropic medication were included.

Seven child characteristics were assessed: (1) gender, (2) age, (3) presence of learning difficulties or a mental disability, (4) general functioning at school, as indicated by a total score based on five self-constructed questions that capture satisfaction with school and school attendance; a higher score implies better functioning at school, (5) severity of internalising problems and (6) of externalising problems, both measured by the respective subscales of the parents’ version of the Strength and Difficulties Questionnaire (SDQ; [23, 24]); and (7) callous and unemotional traits; as assessed by the parent version of the Inventory of Callous-Unemotional Traits (ICU) [25, 26], with higher scores on SDQ and ICU indicating more severe problems or traits.

Characteristics of the primary parent included four predictors: (1) gender, (2) age, (3) current mental health (i.e., depressive and anxiety symptoms) as measured by the self-reported Mental Health Index-5 (MHI) [27]; a higher score indicates better mental health and (4) prior or current help for mental health problems, by asking whether the primary parent received some kind of help for mental health problems, currently or in the past.

Parenting characteristics were assessed with eight measures. Five subscales of the Alabama Parenting Questionnaire (APQ) [28, 29] were used: (1) involved parenting, (2) positive parenting, (3) poor monitoring, (4) inconsistent disciplining and (5) corporal punishment. Furthermore, (6) the General Self-Efficacy scale (GSE) [30] was used to measure the primary parent’s perceived general self-efficacy; a higher score reflects a higher level of perceived general self-efficacy, (7) perceived parenting competence was measured by the Parenting Sense of Competence scale (PSOC) [31]; a higher score indicates a higher level of perceived parenting competence and (8) parental stress of the primary parent was assessed with the Parental Stress Scale (PSS) [32]; a higher score points to more parental stress.

 Family characteristics were investigated by eight family factors: (1) single parent household, (2) presence of other children in the household, (3) socio-economic status, as assessed through a standardised composite score of the net income level of the household and the educational and occupational level of both parents (data from the secondary parent was also used) [33], based on the International Standard Classification of Occupations [34], (4) presence of financial problems, by asking the primary parents whether the monthly net income was sufficient (yes/no/refused to answer), (5) urbanicity, based on the postal code through which participants were classified as living in a small-sized city (< 40,000 inhabitants), medium-sized city (40,000–100,000 inhabitants) or large-sized city (> 100,000 inhabitants), (6) social network strength; defined by a mean composite score of seven standardised variables (e.g., number of contacts with friends and family, frequency of the contact); a higher score points to a stronger social network strength, (7) families with high-risk behaviours; assessed through a composite score of 22 ‘risk’ variables (e.g., use of drugs, parental psychopathology, parental criminal record), a higher score indicating more high-risk behaviour of the family and (8) family functioning, using the Dutch Parental Questionnaire Family Functioning (VGFO) [35]; a higher score on the VGFO indicates a better family functioning regarding basic care for the child, nurture, social contacts of the primary parent, own youth experiences and partner relation of the primary parent.

### Statistical analysis

To investigate which variables were associated with parental pre-treatment motivation, we applied the least absolute shrinkage and selection operator (LASSO) [36] using the *glmnet*-package in R [37, 38]. This method selects the best fitting predictors by automatically assigning a penalized term to the standardised predictors. The selection of the penalty term is done by cross-validation, where the average mean cross-validated error is calculated through 10,000 iterations to get the best fitting penalty term. Since LASSO automatically assigns the penalty term to the predictors with a low error, this method is suitable for selecting the best set of predictors out of a large model. Assumptions of linearity, independence and constant variance of residuals were not violated. Prior to analysis, 15 missing cases on urbanicity (2.88%) were imputed with 5 imputations by applying predictive mean matching via the *mice*-package in R [38, 39]. Furthermore, we tested for outliers with the Mahalanobis distance [40]; no outliers were found.

Six models were run to identify the best fitting predictors for parental treatment motivation. We started with five separate models on our five predictor domains: (1) source of referral and prior use of healthcare services, (2) child characteristics, (3) characteristics of the primary parent, (4) parenting characteristics of the primary parent and (5) family characteristics. In the final overall model, all statistically relevant predictors were included. Statistical relevance of a predictor was defined as the presence of a non-zero beta coefficient and non-zero explained deviance (comparable to explained variance, but based on the − 2 log-likelihood instead of the residuals of the model). The explained deviance of the predictors is calculated through the leave-one-out method; the predictor of interest is left out of the total model causing a change in the explained deviance of the total model. The difference between the explained deviance of the total model and the model without the predictor of interest is the contribution of this predictor to the model [41].

## Results

### Sample characteristics

Table 1 shows the descriptive statistics of all study variables. Ethnicity of the primary parent was not included as a predictor due to the homogeneous sample; 97.1% of primary parents were Dutch. Mothers generally were the primary parents (85%). The mean age of the children was 10.2 years (range 1–18 years) and about 60% were boys. About one-fifth of the children lived in a single parent household. Approximately, 13% of the families were from a low socio-economic background and almost half of the families resided in a small-sized city (< 40,000 inhabitants). Furthermore, about half of the children had received some kind of professional help for their problems in the past and one-fifth of the children currently used medication.

**Table 1:** Descriptive statistics of all study variables obtained from the primary parent (*n* = 521)

	Mean, *n*	Standard deviation, %	Range
Outcome measure			
Treatment motivation of the primary parent (PMI)	3.80	0.53	1–5
Source of referral and prior use of healthcare services			
Source of referral			
Parent(s) or child	326	62.6	
School	89	17.1	
Health professional	106	20.3	
Prior use of mental health services for the child	235	45.1	
Current medication use by the child	110	21.1	
Child characteristics			
Female gender	207	39.7	
Age (years)	10.2	3.76	1–18
Presence of learning difficulties or mental disability	200	38.4	
General functioning at school^a^	3.93	0.75	1–5
Severity of internalising problems (SDQ)	1.80	0.39	1–3
Severity of externalising problems (SDQ)	1.91	0.41	1–3
Callous and unemotional traits (ICU)	2.27	0.43	1–4
Characteristics of the primary parent			
Female gender	443	85.0	
Age (years)	41.2	6.47	25–65
Current mental health (MHI-5)^a^	2.34	0.83	1–6
Prior or current help for mental health problems of the primary parent	255	48.9	
Parenting characteristics of the primary parent			
Involved parenting (APQ)^a^	3.87	0.45	1–5
Positive parenting (APQ)^a^	3.96	0.50	1–5
Poor monitoring (APQ)	2.38	0.43	1–5
Inconsistent disciplining (APQ)	2.47	0.54	1–5
Corporal punishment (APQ)	1.57	0.45	1–5
Perceived general self-efficacy (GSE)^a^	3.08	0.58	1–4
Perceived parenting competence (PSOC)^a^	4.29	0.66	1–5
Parental stress (PSS)	2.03	0.49	1–6
Family characteristics			
Single parent household	113	21.7	
Presence of other children in the household	439	84.3	
Socio-economic status of the family^b^			
Low	67	12.9	
Intermediate	391	75.0	
High	63	12.1	
Presence of financial problems			
Yes	367	70.4	
No	109	20.9	
Refused to answer	45	8.6	
Urbanicity			
Small-sized city (< 40,000 inhabitants)	239	45.9	
Middle-sized city (40,000–100,000 inhabitants)	160	30.7	
Large-sized city (> 100,000 inhabitants)	122	23.4	
High-risk behaviour families			
Low-risk families	75	14.4	
Normal risk families	365	70.1	
High-risk families	81	15.5	
Family functioning (VGFO)^a^	3.25	0.39	1–4

See Online Resource 1 for psychometric properties of the scales

*PMI* parental motivation inventory [5], *SDQ* Strength and Difficulties Questionnaire [18], *ICU* inventory of callous-unemotional traits [20], *MHI-5* Mental Health Index-5 [22], *APQ* Alabama Parenting Questionnaire [23], *GSE* general self-efficacy [25], *PSOC* Parental Sense of Competence Scale [26], *PSS* Parental Stress Scale [27], *VGFO* Parental Questionnaire Family Functioning [30]

^a^A higher score indicates better functioning, mental health or parenting

^b^Includes data from the secondary parent where available

### LASSO regression

Table 2 shows the explained deviances and beta coefficients for the various predictors of the five domain-specific models and the overall model with all relevant predictors based on LASSO regression analyses. As explained, predictors are statistically relevant if they were not shrunk to zero (i.e., beta coefficient and explained deviance were higher than zero).

**Table 2:** Results of LASSO regression from five predictor domains and an overall model for selection of predictors of parental treatment motivation (*n* = 521)

	Model 1: source of referral and prior use of healthcare services		Model 2: child characteristics		Model 3: characteristics of the primary parent		Model 4: parenting characteristics of the primary parent		Model 5: family characteristics		Overall model	
	Explained deviance (%)	*β*	Explained deviance (%)	*β*	Explained deviance (%)	*β*	Explained deviance (%)	*β*	Explained deviance (%)	*β*	Explained deviance (%)	*β*
Total model	4.00		12.1		2.59		5.93		3.92		21.3	
Source of referral (Ref = parent(s) or child)												
School	1.72	− 0.14									1.11	− 0.11
Health professional	1.42	− 0.12									0.71	− 0.09
Prior use of mental health services for the child	0.86	0.09									0.14	0.04
Current medication use by the child	0.39	− 0.06									0.77	− 0.09
Child of female gender			0.00	0.00								
Age of the child (years)			1.49	− 0.12							1.17	− 0.15
Presence of learning difficulties or mental disability of the child			0.10	− 0.02							0.07	− 0.03
General functioning at school of the child^a^			0.00	0.00								
Severity of child’s internalising problems (SDQ)			4.77	0.21							3.25	0.19
Severity of child’s externalising problems (SDQ)			1.55	0.14							0.84	0.11
Callous and unemotional traits of the child (ICU)			0.77	0.08							0.46	0.08
Primary parent of female gender					0.00	0.00						
Age of the primary parent (years)					0.52	− 0.06					0.20	0.06
Current mental health of the primary parent (MHI-5)^a^					0.66	0.08					0.06	0.03
Prior or current help for mental health problems of the primary parent					0.56	0.07					0.46	0.07
Involved parenting of the primary parent (APQ)^a^							0.01	0.00				
Positive parenting of the primary parent (APQ)^a^							0.01	0.00				
Poor monitoring of the primary parent (APQ)							0.12	− 0.01			0.02	− 0.01
Inconsistent disciplining of the primary parent (APQ)							0.01	0.00				
Corporal punishment of the primary parent (APQ)							0.35	− 0.04			0.19	− 0.04
Perceived general self-efficacy of the primary parent (GSE)^a^							1.78	0.11			1.50	0.15
Perceived parenting competence of the primary parent (PSOC)^a^							2.87	− 0.22			1.42	− 0.17
Parental stress of the primary parent (PSS)							0.01	0.00				
Single parent household									1.12	− 0.08	0.67	− 0.09
Presence of other children in the household									0.06	0.01	0.19	0.05
Socio-economic status of the family^b^									0.003	0.00		
Presence of financial problems (Ref = No)												
Yes									2.44	− 0.12	1.57	− 0.15
No answer									1.86	− 0.10	1.09	− 0.12
Urbanicity (Ref = small-sized city (up to 40,000 residents)												
Medium-sized city (40,001–100,000 residents)									0.003	0.00		
Large-sized city (100,001 or more residents)									0.003	0.00		
Social network strength of the primary parent									0.003	0.00		
High-risk behaviour families									0.003	0.00		
Family functioning (VGFO)^a^									0.05	− 0.01	0.47	0.08

See Online Resource 1 for psychometric properties of the scales

*Ref* reference category for dummy variable, *PMI* Parental Motivation Inventory [5], *SDQ* Strength and Difficulties Questionnaire [18], *ICU* inventory of callous-unemotional traits [20], *MHI-5* Mental Health Index-5 [22], *APQ* Alabama Parenting Questionnaire [23], *GSE* general self-efficacy [25], *PSOC* Parental Sense of Competence Scale [26], *PSS* Parental Stress Scale [27], *VGFO* Parental Questionnaire Family Functioning [30]

^a^A higher score indicates a better functioning, mental health or parenting

^b^Includes data from the secondary parent where available

#### Source of referral and prior use of healthcare services

Model 1 showed a total explained deviance of 4.0%. All four variables were relevant predictors in the model; source of referral contributed the most. Both school (1.72%; *β* =  − 0.14) and a health professional (1.42%; *β* =  − 0.12) as source of referral were related to a lower level of parental pre-treatment motivation, in comparison to self-referral by parent(s)/child. Moreover, current use of medication by the child was related to a lower level of parental motivation (0.39%; *β* =  − 0.06). Lastly, prior use of mental health services for the child was related to a higher level of parental motivation (0.86%; *β* = 0.09).

#### Child characteristics

The total explained deviance of Model 2 was 12.1%. Five of the seven variables were relevant predictors of parental pre-treatment motivation. Severity of internalising problems (4.77%; *β* = 0.21) was the strongest contributor to the model, followed by externalising problems (1.55%; *β* = 0.14) and callous and unemotional traits (0.77%; *β* = 0.08), all related to a higher level of parental pre-treatment motivation. In contrast, child’s older age (1.49%; *β* =  − 0.12) and the presence of learning difficulties or a mental disability (0.10%; *β* =  − 0.02) were related to a lower level of parental motivation. However, gender of the child and general functioning at school were non-relevant factors with both beta values and explained deviances shrunk to zero.

#### Characteristics of the primary parent

Model 3 showed a total explained deviance of 2.59%. Three of the four characteristics of the primary parent remained relevant in the model, namely (1) parent’s age, (2) current mental health and (3) prior or current help for mental health problems. The strongest predictor in the model was a better current mental health of the primary parent (0.66%; *β* = 0.08), followed by receiving prior or current help for mental health problems of the primary parent (0.56%; *β* = 0.07), all related to a higher level of parental pre-treatment motivation. In contrast, older primary parents had a lower level of pre-treatment motivation (0.52%; *β* =  − 0.06), while the primary parent’s gender was not a relevant predictor.

#### Parenting characteristics of the primary parent

Model 4 showed a total explained deviance of 5.93%. Four of the eight predictors were relevant predictors for parental pre-treatment motivation: the strongest predictor was a higher perceived parenting competence (*β* =  − 0.22) related to a lower level of parental motivation and explaining 2.87% of its deviance. Furthermore, a higher level of corporal punishment (0.35%; *β* =  − 0.04) and poor monitoring (0.12%; *β* =  − 0.01) were related to a lower level of parental motivation. In contrast, a higher perceived general self-efficacy emerged as the second important predictor, which was related to a higher level of parental pre-treatment motivation (1.78%; *β* = 0.11). Four predictors were not relevant predictors, namely (1) involved parenting, (2) positive parenting, (3) inconsistent disciplining and (4) parental stress. These predictors explained a small percentage of deviance in pre-treatment motivation, most likely due to a shared contribution of the different predictors to the model.

#### Family characteristics

The predictors of the family characteristics domain explained a total of 3.92% of the deviance, with in total five out of ten predictors being relevant. The strongest relation was found for the presence of financial problems. Both the presence of financial problems (2.44%; *β* =  − 0.12) and those who refused to answer whether there were financial problems (1.86%; *β* =  − 0.10) were related to a lower parental pre-treatment motivation, in comparison to the absence of financial problems. Moreover, having a single parent household (1.12%; *β* =  − 0.08) and better family functioning (0.05%; *β* =  − 0.01) were related to a lower level of parental motivation. In contrast, the presence of other children in the household was related to a higher parental motivation (0.06%; *β* = 0.01). Non-relevant factors were: (1) socio-economic status, (2) urbanicity, (3) social network strength and (4) high-risk behaviour families; although a small portion of explained deviance was found, this is most likely due to the shared contribution of different predictors to the model.

#### Overall model

All statistically relevant predictors of the five different predictor domains were entered into one overall model. In total, 21 predictors were entered and all remained relevant, explaining a total of 21.3% of primary parents’ pre-treatment motivation. To compare the relative strength of the different domains, we summed the explained deviances of the individual predictors per domain. We found the highest explained deviance for child characteristics (5.79%), followed by family characteristics (3.99%), parenting characteristics of the primary parent (3.13%), source of referral and prior use of healthcare services (2.79%) and, lastly, characteristics of the primary parent (0.79%).

The strongest predictor related to a higher parental pre-treatment motivation was the severity of the child’s internalising problems (3.25%; *β* = 0.19), and to a lesser extent the externalising behavioural domain (i.e., externalising problems and higher callous–unemotional traits together explaining 1.30%), followed by perceived general self-efficacy of the primary parent (1.50%; *β* = 0.15). In contrast, the strongest predictor related to a lower parental pre-treatment motivation was families’ financial problems and refusal to answer whether there were financial problems (together explaining 2.66%). This was followed by referral to the outpatient clinic prompted by a school or health professional (together explaining 1.82%) rather than by the parent or child, a higher perceived parenting competence (1.42%; *β* =  − 0.17) and an older age of the child (1.17%; *β* =  − 0.15). For other predictors we refer to Table 2.

In the overall model, two factors were noted to change in direction of effects, i.e., higher age of the primary parent (Model 3: 0.52%; *β* =  − 0.06) and better family functioning (Model 5: 0.05%; *β* =  − 0.01) were no longer related to a lower level of parental motivation, but to a higher level of motivation (0.20%; *β* = 0.06 and 0.47%; *β* = 0.08, respectively).

## Discussion

The aim of this study was to identify factors related to parents’ pre-treatment motivation of children and adolescents who had been newly referred to an outpatient child and adolescent mental health clinic. For any treatment to be successful, there is a large dependency on the parents’ willingness to participate in treatment and their readiness to change their own behaviour; regarding behavioural interventions, but also daily organization and possible lifestyle changes [1, 6, 8, 9]. To our knowledge, this is the first study that investigated a large set of individual and contextual factors in relation to parental motivation before the start of treatment across five domains using LASSO regression. We assessed the role of (1) source of referral and prior use of healthcare services, (2) child characteristics, (3) characteristics of the primary parent, (4) parenting characteristics of the primary parent and (5) family characteristics. Notably, our overall model was able to explain more than one-fifth of the primary parent’s pre-treatment motivation based on these individual and contextual variables. While we were able to explain a sizeable portion of parents’ motivation, it should be noted that a total of 21 individual variables emerged as relevant factors from the model, each of them showing a small contribution. This points to a large heterogeneity in factors that may affect parents’ motivation and highlights its multifactorial nature. Child characteristics contributed the most, while, surprisingly, characteristics of the primary parent explained little of parental pre-treatment motivation. The most important factors were the severity and type of the child’s problems, financial problems of the family, source of referral to the clinic, the perceived self-efficacy of the primary parent, parenting competence of the primary parent and age of the child. In the following, we will discuss only the most relevant factors (explained deviance ≥ 1%).

We started by investigating factors related to the source of referral and child’s prior use of mental healthcare. Our study indicates that parents’ pre-treatment motivation is higher when family members themselves decided to reach out for help, rather than when school or health professionals were the primary source of referral. Especially, when the child was referred by the school, the parental motivation to start treatment was lower. An explanation might be that problems arising at school might not be present or apparent at home and, thus, a parent disagrees with the school’s view that their child is in need of behavioural treatment of medication for emotional or behavioural problems.

Regarding child characteristics, the severity of a child’s problems was most strongly linked to parental pre-treatment motivation. This is consistent with previous findings [18–20] where higher symptom severity was related to a higher parental motivation to start treatment. Notably, we found the strongest relation for internalising problems, while the main focus of previous studies was on externalising problem behaviour [19, 20]. Our finding is somewhat surprising since our measure of parental motivation focusses on actively changing a parent’s own behaviour, which appears to be more relevant to the child's externalising than to internalising problems. We assume that a child’s internalising problems may perhaps be more threatening or alarming to parents than a child’s externalising problems and callous–unemotional traits. Parents may also feel a greater sense of helplessness and lack of understanding of their child’s anxious and depressive symptoms. These aspects may increase the willingness to receive treatment and change parental behaviour. We are not aware of existing studies that investigated this link, which makes it an interesting topic for future research. In contrast, parents of older children or adolescents had a lower level of pre-treatment motivation, likely reflecting the normal developmental course with lower involvement from parents of older children or adolescents and the greater availability of therapeutic interventions tailored primarily for child or adolescent participation.

Subsequently, we considered characteristics of the primary parent. In contrast to Fosco et al. [17], we found that parents with a better current mental health showed higher pre-treatment motivation. This suggests that parents with a lesser degree of depressive or anxiety symptoms are more capable to support their child in need of mental health care. A possible explanation for this difference in findings is that we did not specifically look at a certain type of problem, while the abovementioned authors only looked at children with antisocial behaviour.

With respect to parenting characteristics of the primary parent, surprisingly, we found that higher perceived parenting sense of competence was related to a lower level of parents’ pre-treatment motivation, while a higher perceived self-efficacy (e.g., capability of coping with a variety of common demands in life) of the primary parent was associated with a higher treatment motivation. This may appear somewhat contradictory, since parenting sense of competence can be seen as a part of general self-efficacy. An explanation might be that parents who believe that they are competent parents are less inclined to change their parental behaviours due to treatment, while parents with an overall high self-efficacy do want change to happen and believe they can contribute to their child’s treatment. We expected parenting style to be of influence on parental motivation as well. For instance, Andrade et al. [20] found higher levels of inconsistent disciplining and poor monitoring to be related to a higher level of parental motivation. We did not find a strong relation and it seems more likely that poorer parenting skills are related to a lower level of motivation. Furthermore, our study did not confirm a role for parenting stress previously shown to be related to a higher level of parental pre-treatment motivation [18, 19], perhaps explained by the large number of variables in our model that may have been more important (e.g., perceived self-efficacy, perceived parenting competence).

In our final domain, we investigated family characteristics. We found a notable association between presence of financial problems (and refusal to answer that question) and lower level of parents’ pre-treatment motivation but not, as expected, with socio-economic status (based on family income, educational level and occupation of the primary parent and the partner). Furthermore, single parent household contributed, to a lesser extent, to lower parental motivation, independent from financial problems. It thus appears that the broader domain indicating socio-economic position was more important in this model than the classic composite score indicating socio-economic status. Moreover, better family functioning and more children in the household were related to higher parental pre-treatment motivation, however, effects were minor. In contrast to our expectations, social support and high-risk family status (e.g., parental substance use, contact with judicial system) were unrelated to parental pre-treatment motivation.

Strengths of our study were the use of LASSO regression to investigate a wide variety of potential factors of parental pre-treatment motivation, using a well-validated measure, in a large sample of parents and their children newly referred to an outpatient child and adolescent mental health care setting. Although LASSO regression has the limitation that it randomly selects one predictor if factors are correlated [42], we minimized this by running the model 10,000 times to select the best error term in a similar way to the stability selection technique described by Meinshausen and Bühlmann [43]. A limitation might be selection bias; due to the lack of data on families who did not respond to our anonymized study invitation, we were unable to investigate this. Study participants will most likely show higher levels of motivation for treatment than the average referred family at a mental health clinic who did not participate in the study. Therefore, factors that are related to low motivation may be underestimated in our study. Findings should also be considered in light of our study population including a wide variety of mental health problems of varying intensity and may not readily apply to speciality clinics focusing on more severely affected patient groups. However, by including two large child and adolescent psychiatric outpatient centres with different locations, we ensured a large catchment area covering about one-third of the Netherlands. Furthermore, our sample consisted largely of parents of Dutch origin, hence findings may not be generalisable to other ethnicities or minority groups. Finally, we acknowledge that youths’ motivational factors, not addressed in our study, are also an important area of research.

We conclude that mainly child characteristics (severity and type of problems), socio-demographic factors (financial problems and referral through school or health professionals) and parenting characteristics (perceived self-efficacy and parenting competence) are important factors of parental pre-treatment motivation. Since parents’ readiness to participate in treatment and motivation to bring about change are essential in facilitating successful treatment, health professionals should pay particular attention to these individual and contextual factors early during clinical counselling of newly referred patients in a child and adolescent outpatient setting. This will help in identifying particularly vulnerable families characterised by lower motivation for treatment, setting off more targeted strategies to increase treatment motivation in those families. Future research is needed to see how contextual factors are related to response to treatment and how treatment motivation is related to other barriers (e.g., stigma, therapy related problems) in the help-seeking process and during the course of treatment in child and adolescent mental health care.

## Electronic supplementary material

Below is the link to the electronic supplementary material.

Supplementary file 1 (DOCX 36 kb)
