# Supplementary material for: Factors related to parental pre-treatment motivation in outpatient child and adolescent mental health care
Source: Eur Child Adolesc Psychiatry. 2019 Sep 24;29(7):947–58. doi: 10.1007/s00787-019-01391-9 (PMC7321896; doi:10.1007/s00787-019-01391-9)
Supplement: Supplementary file 1 — (DOCX 36 kb) [file 787_2019_1391_MOESM1_ESM.docx]

**Online Resource 1** Description of scales used in this study

| Measure | Reference | Number of items (range of scale items) | Scale description and item samples | Cronbach’s α in validated study (Cronbach’s α in this study) | Notes |
| --- | --- | --- | --- | --- | --- |
| *Child characteristics* | | | | | |
| General functioning at school | self-constructed scale | 5 (1=Not at all true; 5=Entirely true) | Measures behavioural functioning at school. Based on five questions: 1) ‘Tries his/her best at school’; 2) ‘Never skips a class’; 3) ‘Always does his/her homework’; 4) ‘Stays home often due to illness’ and 5) ‘Enjoys going to school’. | (0.73) |  |
| Strength and Difficulties Questionnaire (SDQ): internalising and externalising subscale | (Goodman, 1997) | 25 (1=Not true; 3=Certainly true). | Addresses emotional and behavioural symptoms in the past six months. Item examples are ‘Considerate of other people’s feelings’; ‘Often has temper tantrums or hot tempers’; ‘Easily distracted, concentration wanders’; and ‘Often lies or cheats’. | Internalising: 0.84-0.87 (0.68)  Externalising: 0.87-0.93 (0.78) | The internalising scale consists of 10 items (mean of the ‘emotional problems’ and ‘peer problems’ subscales). The externalising scale consists of 10 items (mean of the ‘Conduct problems’ and ‘Hyperactivity’ subscales). |
| Inventory of Callous-Unemotional Traits (ICU) | (Frick, 2004; Roose, Bijttebier, Decoene, Claes, & Frick, 2010) | 24 (1=Not at all true; 4=Definitely true) | Measures callous, uncaring and unemotional traits, for example, ‘Expresses his/her feelings openly’; ‘Does not care about being on time’; ‘Always tries his/her best’. | 0.83 (0.87) |  |
| *Characteristics of the primary parent* | | | | | |
| Mental Health Index-5 (MHI) | (Berwick et al., 1991) | 5 (1=Never; 6=Always) | Addresses parental mental health in the past month; depressive symptoms, anxiety, burn-out. Item examples: ‘How much of the time, during the last month, have you been a very nervous person?’; ‘How much of the time, during the last  month, have you felt calm and peaceful?’; ‘How much of the time, during the last month, have you been a happy person?’ | 0.79-0.88 (0.86) |  |

*(Continued)*

**Online Resource 1** (Continued)

| Measure | Reference | Number of items (and scale) | Scale description and item samples | Cronbach’s α in validated study (and Cronbach’s α in this study) | Notes |
| --- | --- | --- | --- | --- | --- |
| *Parenting characteristics of the primary parent* | | | | | |
| Alabama Parenting Questionnaire (APQ) | (Frick, 1991) | 42 (1=Never; 5=Always) | Measures five dimensions of parenting, namely 1) involved parenting, 2) positive parenting, 3) poor monitoring, 4) inconsistent disciplining and 5) corporal punishment. Example items: ‘You have a friendly talk with your child.’; ‘Your child fails to leave a note or to let you know where he/she is going.’; ‘You feel that getting your child to obey you is more trouble than it’s worth.’ | Positive parenting:0.79-0.82  Parental involvement: 0.74-0.81  Poor monitoring/supervision: 0.81-0.83  Corporal punishment: 0.79-0.83  Inconsistent disciplining: 0.54-0.62 | The five subscales were used separately in the study. |
| General Self-Efficacy (GSE) | (Schwarzer & Jerusalem, 1995) | 10 (1=Not at all true; 4=Exactly true) | Measures general self-efficacy. Example items: ‘I can always manage to solve difficult problems if I try hard enough’; ‘I can remain calm when facing difficulties because I can rely on my coping abilities.’; ‘I can usually handle whatever comes my way.’ | 0.76-0.90 (0.91) |  |
| Parental Stress Scale (PSS) | (Berry & Jones, 1995) | 18 (1=Strongly disagree; 5=Strongly agree) | Assesses stress with parenting. Example items: ‘I am happy in my role as a parent’; ‘Having children has been a financial burden’; ‘I feel overwhelmed by the responsibility of being a parent’. | 0.83 (0.85) |  |
| Parenting Sense of Competence Scale (PSOC) | (Johnston & Mash, 1989) | 16 (1=Strongly disagree; 6=Strongly agree) | Measures parenting competence. Example items are ‘My mother/father was better prepared to be a good mother/father than I am.’; ‘Being a parent is manageable, and any problems are easily solved.’; ‘Sometimes I feel like I’m not getting anything done’ | 0.58-0.82 (0.82) |  |

**Online Resource 1** (Continued)

| Measure | Reference |  | Item number (and scale) | Item samples | | Notes |
| --- | --- | --- | --- | --- | --- | --- |
| High risk behaviour Families | Categorisation based on self-constructed scale |  | Yes/no | Do you own a car? | | High risk behaviour families is calculated by the average mean of the 22 standardised variables. |
|  |  |  | Yes/no | Sufficient monthly net income | |  |
|  |  |  | Yes/no | Financial troubles now | |  |
|  |  |  | Yes/no | Financial troubles in the past | |  |
|  |  |  | 0-40 glasses per week | Alcohol use primary parent | |  |
|  |  |  | 0-40 glasses per week | Alcohol use secondary parent | |  |
|  |  |  | Yes/no | Drug use primary parent now | |  |
|  |  |  | Yes/no | Drug use primary parent in the past | |  |
|  |  |  | Yes/no | Drug use secondary parent now | |  |
|  |  |  | Yes/no | Drug use secondary parent in the past | |  |
|  |  |  | Yes/no | Child came into contact with judicial system (last six months) | |  |
|  |  |  | Yes/no | Child came into contact with judicial system (in the past) | |  |
|  |  |  | Yes/no | Lawsuit (past six months) | |  |
|  |  |  | Yes/no | Lawsuit (in the past) | |  |
|  |  |  | Yes/no | Court custody of the child (in the past) | |  |
|  |  |  | Yes/no | Came into contact with judicial system (in the past six months) | |  |
|  |  |  | Yes/no | Ever arrested (past six months) | |  |
|  |  |  | Yes/no | Ever arrested (in the past) | |  |
|  |  |  | Yes/no | Criminal record primary parent | |  |
|  |  |  | Yes/no | Criminal record secondary parent | |  |
|  |  |  | Yes/no | Psychopathology primary parent | |  |
|  |  |  | Yes/no | Psychopathology secondary parent | |  |
| Parental Questionnaire Family Functioning (VGFO) | (Janssen & Veerman, 2006) |  | 30 (1=Not true; 4=Entirely true) | Addresses five dimensions of family functioning, namely 1) basic care, 2) nurturing, 3) social contacts, 4) own youth experiences and 5) relationship with partner. Example items: ‘I give my children enough freedom’; ‘I have had a great childhood’; ‘my parents had too little time for me’ | 0.90 (0.89) |  |
